# Editorial: Beyond CpG Methylation: New Modifications in Eukaryotic DNA

**DOI:** 10.3389/fcell.2018.00087

**Published:** 2018-08-07

**Authors:** Reinhard Stöger, Alexey Ruzov

**Affiliations:** ^1^School of Biosciences, University of Nottingham, Nottingham, United Kingdom; ^2^Division of Cancer and Stem Cells, School of Medicine, University of Nottingham, Nottingham, United Kingdom

**Keywords:** DNA modifications, 5-methylcytosine, 5-hydroxymethylcytosine, 5-formylcytosine, 5-carboxylcytosine, N6-methyldeoxyadenosine

“***Modification—[mod-uh-fi-key-shuh n]***”: Numerous definitions exist for this word, but they all include descriptions that fit the theme covered in this collection of articles: “….*the making of a limited change in something*…”; “…*a limitation or qualification of the meaning of a word by an affix*..” “…*a change in an organism caused by environmental factors*…”; “…*transformation from its original form during development or evolution…*.”

Hosted by the journals *Frontiers in Cell and Developmental Biology and Frontiers in Genetics* the compilation of papers examines modifications that can confer new meaning to some of the four canonical letters in the DNA alphabet. The focus is on biologically relevant DNA modifications known to exist at the present in genomes of higher eukaryotes.

Since reporting in 2009 that 5-methyldeoxycytosine (5mC) is not the only covalent DNA modification present in biologically relevant quantities in animal genomes, there has been a flurry of publications describing, measuring, and contemplating the functions of these epigenetic marks—too many to keep abreast with, even if you are fascinated by this research field.

The contributing papers to this research topic provide a snapshot in time of our current understanding of DNA modifications; by no means is it comprehensive. However, if you aim to gain insight on certain aspects of DNA modifications, you may find it here.

The idea that epigenetic information has the potential to be inherited has captured the attention of the scientific community. Thus, everything you always wanted to know about sex, or more specifically, about the mammalian sperm epigenome can be explored in a timely review by Champroux et al.

5mC can be oxidized to 5-hydroxymethyldeoxycytosine (5hmC), 5-formyldeoxycytosine (5fC), and 5-carboxyldeoxycytosine (5caC). These oxidized cytosine modifications are catalyzed by “10-11 translocation methylcytosine dioxygenases”—in short, TETs. But where are TETs expressed and why do cells generate isoforms of the three known TET genes in mammals? The concise short review by Melamed et al. summarizes what is known about these enzymes.

An abundance of 5hmC is present in the brain and this DNA modification appears to be very responsive to environmental and physiological changes. Jessop and Toledo-Rodriguez communicate that voluntary exercise halts the decline of age-related expression of *TET1* and *TET2*, genes encoding enzymes that catalyse the oxidation of 5mC to 5hmC in the hippocampus.

Patterns of DNA modifications are very dynamic in preimplantation embryos and in primordial germ cells. The potential functions of all known mammalian DNA modifications in epigenetic inheritance through the germ line, both male and female, is comprehensively reviewed and discussed by Zhu et al.

Our understanding of epigenetics in human disease has been strongly influenced by studies of cancer. Ramsawhook et al. build on this rich history and explore the interplay between TET enzymes and the Wilms' Tumor protein 1 (WT1) in brain tumors.

Oxidized forms of 5mC, including 5fC and 5caC have been detected in many species. Is their spatio-temporal distribution in different genomes the same? Jessop et al. compare and evaluate the roles these cytosine modifications and TET proteins play in two widely used model organisms—the mouse and the zebrafish.

Regulation of DNA modifications has a pronounced influence on the state of undifferentiated cells—pluripotent and primordial germ cells. The mini-review by Seki highlights the importance of PRDM14, a DNA binding protein, which stabilizes transcriptional networks in pluripotent cells.

We are confident that you will find stimulating facts and ideas in this set of articles, which will complement your knowledge and help drive your research—enjoy.

## Author contributions

All authors listed have made a substantial, direct and intellectual contribution to the work, and approved it for publication.

### Conflict of interest statement

The authors declare that the research was conducted in the absence of any commercial or financial relationships that could be construed as a potential conflict of interest. The handling editor declared a shared affiliation, though no other collaboration, with the authors.

